# Antileukaemic rescue by dose-dense donor-lymphocyte infusions in T-PLL after allogeneic stem cell transplantation – a case report

**DOI:** 10.1007/s00277-024-05929-z

**Published:** 2024-09-06

**Authors:** Christian Späth, Thomas Neumann, William H. Krüger

**Affiliations:** Dept. Internal Medicine C, Haematology, Oncology, Stem Cell Transplantation, University Hospital Greifswald, Palliative Care. Ferdinand Sauerbruch Street, 17475 Greifswald, Germany

**Keywords:** T-Cell prolymphocytic leukaemia, Allogeneic stem cell transplantation, Donor-lymphocyte infusions, GvL-effects

## Abstract

T-Cell Prolymphocytic Leukaemia (T-PLL) is an aggressive disease with a poor prognosis and only curable by allogeneic stem cell transplantation. We describe the case of a male suffering from T-PLL. Therapy was alemtuzumab followed by an allograft from an unrelated donor. T-PLL relapsed after allogeneic stem cell transplantation. Discontinuation of immunosuppression had no effected and three increasing doses of donor lymphocytes were given within one month. The patient developed acute GvHD of the lover (grade III). GvHD was successfully treated by steroids and ruxolitinib and graft-versus-leukaemia effects induced a complete remission of T-PLL. 18,5 months after transplantation the patient is well and alive without GvHD under immunosuppression with ruxolitinib. Flow cytometry of peripheral blood was negative for residual leukemic cells.

T-Cell Prolymphocytic Leukaemia (T-PLL) is a rare but aggressive disease with a poor prognosis. T-PLL responds poorly to chemotherapy and the standard of care is the monoclonal antibody alemtuzumab. Patients achieving a remission under this therapy should proceed to allogeneic haemopoietic stem cell transplantation. This modality is potentially curative, but with only moderate success [1, 2]. Allogeneic stem cell transplantation is the only curative option for these patients and the contribution of GvL-effects to curing have been described, however, relapses are not rare [3–5].

Patient case: T-PLL was diagnosed in a 65-year-old male in October 2022. He presented with leucocytosis (51Gpt/l), Anaemia (5,3mmol/l), thrombopenia (51Gpt/l). Bone marrow examination showed a 65% infiltration with T-PLL cells. Flow-cytometry detected 72% T-PLL cells in marrow. Additionally, he had pericardial and bilateral pleural effusions and ascites without detection of malignant cells in puncture samples. Cytogenetical and moleculargenetical examinations of T-PLL cells revealed a paracentric inversion of the long arm of chromosome 14. Aberrations of TP53 were not detected. Molecular genetic examinations revealed the TRA/D and TCL1A rearrangements typical for T-PLL.

Co-morbidities were a coronary heart disease and diabetes mellitus type 2. Treatment of T-PLL was initiated with 100 mg prednisolone over 5 days followed by alemtuzumab 30 mg three times per week. A cytological and histological remission was reached after 5 weeks, however, flow-cytometry detected still 0,11% leukaemic cells. Since further five weeks of alemtuzumab did not further improve the depth of remission, the patient proceeded to allogeneic stem cell transplantation. After conditioning with 8 Gy TBI, 120 mg/m² fludarabine and 10 mg alemtuzumab he was allografted with a non-manipulated graft (peripheral blood stem cells, CD34^+^-cells: 3,04*10^6^/kg recipient weight) from a 10/10 antigen matched female unrelated donor [6]. GvHD-prophylaxis was cyclosporine-A and short-course MTX. Leukocytes engrafted at day + 23 and platelets at day + 29. Bone marrow examinations at days + 31 and + 59 revealed cytologically and histologically a complete remission, however, flow-cytometry detected 0,32% and 0,61% residual T-PLL cells, respectively.

Immunosupression was discontinued at day + 62 after stem cell transplantation. Donor-lymphocytes were ordered and infusions were initiated. 5*10^5^, 10^6^, and 5*10^6^ CD3^+^ T-cells per kilogram bodyweight were infused at days + 110, +123, and + 138 after allogeneic stem cell transplantation. The patient developed acute GvHD (skin 0, gut 0, liver 3, overall grade 3). The GvHD of the liver was proven histologically. Furthermore, the liver histology gave no evidence for an EBV-hepatitis. Other forms of viral hepatitis (A, B, C, E) were excluded by serology. The GvHD was successfully treated with steroids, cyclosporine-A and ruxolitinib.

Bone marrow examination at day + 221 was negative for T-PLL cells by cytology, histology and flow-cytometry. Donor chimerism was 98,7% and 98% in blood and marrow, respectively. Steroids and cyclosporine could be successfully withdrawn, however an attempt to taper ruxolitinib failed 13 months after transplantation. Liver GvHD relapsed and responded again to escalated immunosuppression. 18,5 months after transplantation the patient is well and alive without signs of active GvHD under immunosuppression with ruxolitinib 10 mg twice daily. Flow cytometry of peripheral blood was negative for residual leukemic cells.

Dose-dense donor lymphocyte infusions with increasing doses led to acute GvHD and GvL-effects against the leukaemic clone were induced. Smoldering GvHD probable contributes to sustainment of remission, even when clinical signs of GvHD are suppressed. T-PLL is a rare disease only curable by allogeneic haemopoietic stem cell transplantation. GvL-effects and conditioning with protocols containing TBI with ≥ 6 Gy can contribute substantially to curing [7].

## Data Availability

No datasets were generated or analysed during the current study.
